# Neuron’s eye view: Inferring features of complex stimuli from neural responses

**DOI:** 10.1371/journal.pcbi.1005645

**Published:** 2017-08-21

**Authors:** Xin Chen, Jeffrey M. Beck, John M. Pearson

**Affiliations:** 1 Duke Institute for Brain Sciences, Duke University, Durham, North Carolina, United States of America; 2 Center for Cognitive Neuroscience, Duke University, Durham, North Carolina, United States of America; 3 Department of Neurobiology, Duke University Medical Center, Durham, North Carolina, United States of America; Stony Brook University, UNITED STATES

## Abstract

Experiments that study neural encoding of stimuli at the level of individual neurons typically choose a small set of features present in the world—contrast and luminance for vision, pitch and intensity for sound—and assemble a stimulus set that systematically varies along these dimensions. Subsequent analysis of neural responses to these stimuli typically focuses on regression models, with experimenter-controlled features as predictors and spike counts or firing rates as responses. Unfortunately, this approach requires knowledge in advance about the relevant features coded by a given population of neurons. For domains as complex as social interaction or natural movement, however, the relevant feature space is poorly understood, and an arbitrary *a priori* choice of features may give rise to confirmation bias. Here, we present a Bayesian model for exploratory data analysis that is capable of automatically identifying the features present in unstructured stimuli based solely on neuronal responses. Our approach is unique within the class of latent state space models of neural activity in that it assumes that firing rates of neurons are sensitive to multiple discrete time-varying features tied to the *stimulus*, each of which has Markov (or semi-Markov) dynamics. That is, we are modeling neural activity as driven by multiple simultaneous stimulus features rather than intrinsic neural dynamics. We derive a fast variational Bayesian inference algorithm and show that it correctly recovers hidden features in synthetic data, as well as ground-truth stimulus features in a prototypical neural dataset. To demonstrate the utility of the algorithm, we also apply it to cluster neural responses and demonstrate successful recovery of features corresponding to monkeys and faces in the image set.

## Author summary

Many neuroscience experiments begin with a set of reduced stimuli designed to vary only along a small set of variables. Yet many phenomena of interest—natural movies, objects—are not easily parameterized by a small number of dimensions. Here, we develop a novel Bayesian model for clustering stimuli based solely on neural responses, allowing us to discover which latent features of complex stimuli actually drive neural activity. We demonstrate that this model allows us to recover key features of neural responses in a pair of well-studied paradigms.

## Introduction

The question of how the brain encodes information from the natural world forms one of the primary areas of study within neuroscience. For many sensory systems, particularly vision and audition, the discovery that single neurons modulate their firing of action potentials in response to particular stimulus features has proven foundational for theories of sensory function. Indeed, neuronal responses to contrast, edges, and motion direction appear to form fundamental primitives on which higher-level visual abstractions are built. Nevertheless, many of these higher-level abstractions do not exist in a stimulus space with obvious axes. As a result, experimenters must choose *a priori* features of interest in constructing their stimulus sets, with the result that cells may appear weakly tuned due to misalignment of stimulus and neural axes.

For example, in vision, methods like reverse correlation have proven successful in elucidating response properties of some cell types, but such techniques rely on a well-behaved stimulus space and a highly constrained encoding model in order to achieve sufficient statistical power to perform inference [1–3]. However, natural stimuli are known to violate both criteria, generating patterns of neural activity that differ markedly from those observed in controlled experiments with limited stimulus complexity [3–5]. Information-based approaches have gone some way in addressing this challenge [4], but this approach assumes a metric structure on stimuli in order to perform optimization, and was recently shown to be strongly related to standard Poisson regression models [6].

More recently, Gallant and collaborators have tackled this problem in the context of fMRI, demonstrating that information present in the blood oxygen level-dependent (BOLD) signal is sufficient to classify and map the representation of natural movie stimuli across the brain [7–9]. These studies have used a number of modeling frameworks, from Latent Dirichlet Allocation for categorizing scene contents [9] to regularized linear regression [8] to sparse nonparametric models [7] in characterizing brain encoding of stimuli, but in each case, models were built on pre-labeled training data. Clearly, a method that could infer stimulus structure directly from neural data themselves could extend such work to less easily characterized stimulus sets like those depicting social interactions.

A rich body of previous work has addressed the problem of identifying low-dimensional latent dynamics underlying neural firing. Typically, these models assume a continuous latent state governed by a linear dynamical system [10–17]. Using generalized linear models and latent linear dynamical systems as building blocks, these models have proven able to infer (functional) connectivity [10], estimate spike times from a calcium images [11], and identify subgroups of neurons that share response dynamics [13, 16, 17]. Inference in these models is generally performed via expectation maximization, though [14–19] also used a variational Bayesian approach. In each case, the focus has typically been on inferring the dynamics of intrinsic neural activity, perhaps conditioned on known covariates **x**_*t*_. Our work is distinct, however, in focusing on inferring features within *stimuli* that drive repeatable patterns of firing across time and trials.

Our model sits at the intersection of these regression and latent variable approaches. We utilize a Poisson observation model that shares many of the same features as the commonly used generalized linear models for Poisson regression. We also assume that the latent features modulating neural activity are time-varying and Markov. However, we make 3 additional unique assumptions: First, we assume that the activity of each neuron is modulated by a combination of multiple independent latent features governed by Markov dynamics. (This can be extended to the semi-Markov case; see Supplementary Information). This allows for latents to evolve over multiple timescales with non-trivial duration distributions, much like the hand-labeled features in social interaction data sets. Second, we assume that these latents are tied to stimulus presentation. That is, when identical stimuli are presented, the *same* latents are also present. This allows us to selectively model the dynamics of latent features of the *stimulus* that drive neural activity, rather than intrinsic neural dynamics (e.g., variation within and across trials). Finally, we enforce a sparse hierarchical prior on modulation strength that effectively limits the number of latent features to which the population of neurons is selective. This allows for a parsimonious explanation of the firing rates of single units in terms of a small set of stimulus features. Finally, we perform full variational Bayesian inference on all model parameters and take advantage of conditional conjugacy to generate coordinate ascent update rules, nearly all of which are explicit. Combined with forward-backward inference for latent states, our algorithm is exceptionally fast, automatically implements Occam’s razor, and facilitates proper model comparisons using the variational lower bound.

However, as noted above, we are not the first to employ variational Bayesian methods to the problem of inferring latent firing rate states. Moreover, several other models have made use of the idea of discrete latent states and Markov models as explanations of neural dynamics [19, 20]. Both of those methods used a Hidden Markov Model (HMM) to capture variability in neural firing in time and identify discrete modes or states of spiking that could be driven by both spike history and external covariates. In [19], this state space was assumed to be organized according to a binary tree, dramatically reducing model complexity. Our model differs from both of these in assuming that the states that govern firing are *deterministic* functions of stimuli, and that these states are a collection of discrete, independent stimulus features, not a single HMM. Thus, while previous models serve well to capture transitions between discrete states of neural activity, our model discovers statistically reliable patterns of activity that are consistent across repeated presentations of a given stimulus. By directly associating latent factors that drive firing with stimulus features, we thus achieve a means of (multiply) coding a given stimulus. That is, we focus on binary latent states as a means of labeling a finite number of overlapping stimulus features.

Most importantly, as we will show, the stimulus features found by our model are often *interpretable*. The choice to assign multiple discrete, independent tags to each stimulus results in a combinatorial code, with capacity exponential in the number of tags. This can, in principle, accommodate a hierarchical structure (as in [19]), but need not. Yet the ultimate goal of latent state models such as ours is to provide a low-dimensional *description* of neural responses, not simply a compression of them. In practice, experimentalists may perform an initial screening experiment by exposing an organism to a broad range of stimuli, with few fixed hypotheses about responsiveness. A given population of neurons may respond to only a few stimulus features, and features so inferred do not necessarily generalize to new brain structures, nor to stimuli outside the initial set. The value of our model, as with topic models and other latent space models, comes in identifying stimulus features that are readily interpretable: we expect our method will be most useful when the latent tags it identifies group stimuli into useful categories that generate hypotheses for future experiments.

In the sections below, we outline the mathematics behind our model, discuss the process of approximate Bayesian inference we use to infer stimulus features, and perform a series of validation experiments on both synthetic data and real data sets of spiking responses. In the latter, we have chosen datasets where the features that drive spiking are reasonably well understood. We train our model without using this information and then compare the inferred and experimenter-labeled features as a means of illuminating strengths and weaknesses of our model. We conclude by discussing possible extensions and applications to other domains.

## Model

### Observation model

**Fig 1:**
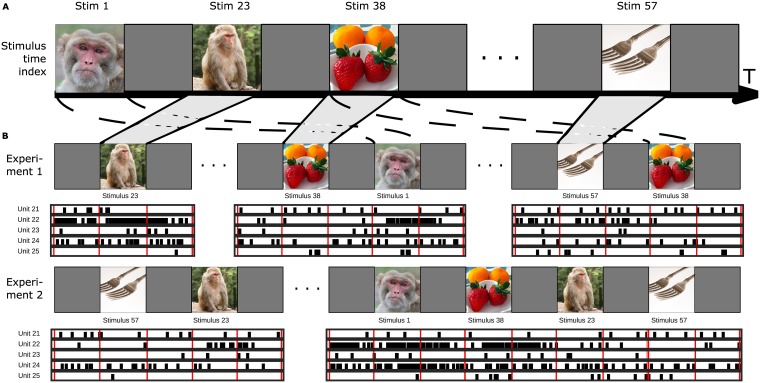
Observational model. A: Stimuli are concatenated to form a single time series indexed by *t*. B: Individual experimental sessions draw from the available set of stimuli, with index *m* representing unique (time, unit) presentations. Example stimulus sequences for two experimental sessions are shown, with corresponding neuronal spike data. Note that the number of presentations of each stimulus can differ by unit, and that units need not be simultaneously recorded. Images copyright Geoff Gallice, (retrieved from Wikimedia Commons), kimumbert/Flickr and dvs/Flickr under CC-BY. Stim 23 image copyright J.M. Garg (used with permission).

Consider a population of *U* spiking neurons or units exposed to a series of stimuli indexed by a discrete time index *t* ∈ {1 … *T*}. We assume that this time index is unique across all stimuli, such that a particular *t* represents a unique moment in a particular stimulus. In order to model repeated presentations of the same stimulus to the same neuron, we further assume that each neuron is exposed to a stimulus *M*_*tu*_ times, though we do not assume any relationship among *M*_*tu*_. That is, we need not assume either that all neurons see each stimulus the same number of times, nor that each stimulus is seen by all neurons. It is thus typical, but not required, that *M*_*tu*_ be sparse, containing many 0s, as shown in Fig 1.

Each unique observation *m* in our data set consists of a spike count *N*_*m*_ for a particular (time, unit) pair (*t*(*m*), *u*(*m*)). We model these spike counts as arising from a Poisson distribution with rate Λ_*tu*_ and observation-specific multiplicative overdispersion *θ*_*m*_:

$$N_{m}∼\text{Pois}(\Lambda_{t(m),u(m)}\theta_{m})\;\;\text{where}\;\;\theta_{m}∼\text{Gamma}(s_{u(m)},s_{u(m)})$$ (1)

That is, for a given stimulus presentation, the spiking response is governed by the firing rate Λ (we set Δ*t* = 1 for convenience), specific to the stimulus and unit, along with a moment-by-moment noise in the unit’s gain, *θ*_*m*_. We restrict these *θ*_*m*_ to follow a Gamma distribution with the same shape and rate parameters, since this results in an expected noise gain of 1. In practice, we model this noise as independent across observations, though it is possible to weaken this assumption, allowing for *θ*_*m*_ to be autocorrelated in time (see Supplementary Information). Note that both the unit and time are functions of the observation index *m*, and that the distribution of the overdispersion for each observation may be specific to the unit observed.

### Firing rate model

At each stimulus time *t*, we assume the existence of *K* binary latent states *z*_*tk*_ and *R* observed covariates *x*_*tr*_. The binary latent states can be thought of as time-varying “tags” of each stimulus—for example, content labels for movie frames—and are modeled as Markov chains with initial state probabilities *π*_*k*_ and transition matrices *A*_*k*_. The observed covariates, by contrast, are known to the experimenter and may include contrast, motion energy, or any other *a priori* variable of interest.

We further assume that each unit’s firing rate at a particular point in time can be modeled as arising from the product of three effects: (1) a baseline firing rate specific to each unit (λ_0_), (2) a product of responses to each latent state (λ_*z*_), and (3) a product of responses to each observed covariate (λ_*x*_):

$$\begin{array}{c} \Lambda_{tu}=\lambda_{0u}\prod_{k=1}^{K}{\left(\lambda_{zuk}\right)}^{z_{tk}}\prod_{r=1}^{R}{\left(\lambda_{xur}\right)}^{x_{tr}} \end{array}$$ (2)

Note that this is conceptually similar to the generalized linear model for firing rates (in which we model log Λ) with the identification *β* = log λ. However, by modeling the firing rate as a product and placing Gamma priors on the individual effects, we will be able to take advantage of closed-form variational updates resulting from conjugacy that avoid explicit optimization (see below). Note also, that because we assume the *z*_*tk*_ are binary, the second term in the product above simply represents the cumulative product of the gain effects for those features present in the stimulus at a given moment in time.

In addition, to enforce parsimony in our feature inference, we place sparse hierarchical priors with hyperparameters *γ* = (*c*, *d*) on the λ_*z*_ terms:

$$\lambda_{zuk}∼\text{Gamma}(c_{zk},c_{zk}d_{zk})\;c_{zk}∼\text{Gamma}(a_{ck},b_{ck})\;d_{zk}∼\text{Gamma}(a_{dk},b_{dk})$$ (3)

That is, the population distribution for the responses to latent features is a gamma distribution, with parameters that are themselves gamma-distributed random variables. As a result, $E\left[\lambda_{u}\right]=d^{-1}$ and var[λ_*u*_] = (*cd*^2^)^−1^, so in the special case of *c* large and d∼O(1), the prior for firing rate response to each latent feature will be strongly concentrated around gain 1 (no effect). As we show below, this particular choice results in a model that only infers features for which the data present strong evidence, controlling for spurious feature detection. In addition, this particular choice of priors leads to closed-form updates in our variational approximation. For the baseline terms, λ_0*u*_, we use a non-sparse version of the same model; for the covariate responses, λ_*xu*_, we model the unit effects non-hierarchically, using independent Gamma priors for each unit.

**Fig 2:**
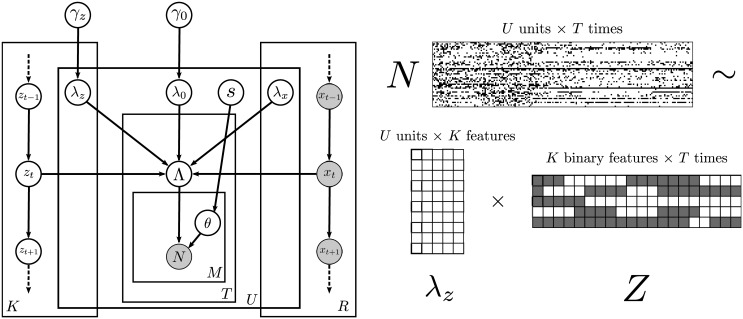
Generative model for spike counts. A: Counts are assumed Poisson-distributed, with firing rates Λ that depend on each unit’s baseline (λ_0_), as well as responses to both latent discrete states *z*_*t*_ (λ_*z*_) and observed covariates *x*_*t*_ (λ_*x*_) that change in time. *γ* nodes represent hyperparameters for the firing rate effects. *θ* is a multiplicative overdispersion term specific to each observation, distributed according to hyperparameters *s*. B: Spike counts *N* are observed for each of *U* units over stimulus time *T* for multiple presentations.

Putting all this together, we then arrive at the full generative model:

p(N,Λ,θ)=p(N|Λ,θ)p(Λ|λ,z)p(λ|γ)p(γ)p(z|A,π)p(A)p(π)p(θ|s)p(s) (4)

where

$$p(\lambda |\gamma )=\prod_{u}p\left(\lambda_{0u}|c_{0},d_{0}\right)\prod_{kr}p\left(\lambda_{zuk}|c_{zk},d_{zk}\right)p\left(\lambda_{xur}\right)$$ (5)

and

$$p(\gamma )=p\left(c_{0}\right)p\left(d_{0}\right)\prod_{k}p\left(c_{zk}\right)p\left(d_{zk}\right)$$ (6)

in conjunction with the definitions of *p*(*N*|Λ, *θ*) and Λ(λ, *z*, *x*) in Eqs (1) and (2). The generative model for spike counts is illustrated in Fig 2.

### Inference

Given a sequence of stimulus presentations (*t*(*m*), *u*(*m*)) and observed spike counts *N*_*m*_, we want to infer both the model parameters Θ = (λ_0_, λ_*z*_, λ_*x*_, *A*, *π*, *c*_0_, *d*_0_, *c*_*z*_, *d*_*z*_, *s*) and latent variables *Z* = (*z*_*kt*_, *θ*_*m*_). That is, we wish to calculate the joint posterior density:

p(Θ,Z|N)∝p(N|Z,Θ)p(Z)p(Θ) (7)

In general, calculating the normalization constant for this posterior is computationally intractable. Instead, we will use a variational approach, approximating *p*(Θ, *Z*|*N*) by a variational posterior *q*(*Z*, Θ) = *q*_*Z*_(*Z*)*q*_Θ_(Θ) that factorizes over parameters and latents but is nonetheless close to *p* as measured by the Kullback-Leibler divergence [21, 22]. Equivalently, we wish to maximize the variational objective

$$\begin{array}{c} L\equiv E_{q}[\log\frac{p(\Theta ,Z|N)}{q(\Theta ,Z)}]=E_{q}[\log p(\Theta ,Z|N)]+H\left[q_{\Theta }\left(\Theta \right)\right]+H\left[q_{Z}\left(Z\right)\right] \end{array}$$ (8)

with H the entropy. We adopt the factorial HMM trick of [23], making the reasonable assumption that the posterior factorizes over each latent time series *z*_⋅*k*_ and the overdispersion factor *θ*_*m*_, as well as the rate parameters *λ*_⋅*u*_ associated with each Markov process. This factorization results in a variational posterior of the form:

$$\begin{array}{c} q\left(\Theta ,Z\right)=q\left(c_{0}\right)q\left(d_{0}\right)\prod_{m}q\left(\theta_{m}\right)\prod_{u}q\left(s_{u}\right)q\left(\lambda_{0u}\right)\prod_{r}q\left(\lambda_{xur}\right)\times \\ \prod_{k}q\left(c_{k}\right)q\left(d_{k}\right)q\left(\lambda_{zuk}\right)q\left(c_{zk}\right)q\left(d_{zk}\right)q\left(z_{k}\right)q\left(\pi_{k}\right)q\left(A_{k}\right) \end{array}$$ (9)

With this ansatz, the variational objective decomposes in a natural way, and choices are available for nearly all of the *q*s that lead to closed-form updates.

### Variational posterior

From Eqs (1) and (2) above, we can write the probability of the observed data *N* as

$$\begin{array}{c} p(N,z|x,\Theta )=\underset{m}{\prod }\left[\frac{{\left(\theta_{m}\Lambda_{t\left(m\right)u\left(m\right)}\right)}^{N_{m}}e^{-\theta_{m}\Lambda_{t\left(m\right)u\left(m\right)}}}{N_{m}!}\right]\underset{mk}{\prod }{\left(A_{k}\right)}_{z_{t\left(m\right)+1,k},z_{t\left(m\right),k}}\underset{k}{\prod }{\left(\pi_{k}\right)}_{z_{0k}} \end{array}$$ (10)

where again, *m* indexes observations of (*t*(*m*), *u*(*m*)) pairs, the portion in brackets is the Poisson likelihood for each bin count and the last two nontrivial terms represent the probability of the Markov sequence given by *z*_*tk*_. From this, we can expand the log likelihood:

$$\begin{array}{c} \log p\left(N,z|x,\Theta \right)=\sum_{mkr}[N_{m}(\log\theta_{m}+\log\lambda_{0u(m)}+z_{t(m)k}\log\lambda_{zu(m)k}+x_{t(m)r}\log\lambda_{xu(m)r})]\\ -\sum_{m}\theta_{m}\Lambda_{t(m)u(m)}+\sum_{mk}\log{\left(A_{k}\right)}_{z_{t(m)+1,k},z_{t(m),k}}+\sum_{k}\log{\left(\pi_{k}\right)}_{z_{0k}}+\text{constant,} \end{array}$$ (11)

Given that Eq (11) is of an exponential family form for *θ* and λ when conditioned on all other variables, free-form variational arguments [21] suggest variational posteriors:

$$\lambda_{0u}∼\text{Gamma}(\alpha_{0u},\beta_{0u})$$ (12)

$$\lambda_{zuk}∼\text{Gamma}(\alpha_{zuk},\beta_{zuk})$$ (13)

$$\lambda_{xur}∼\text{Gamma}(\alpha_{xur},\beta_{xur})$$ (14)

For the first of these two, updates in terms of sufficient statistics involving expectations of *γ* = (*c*, *d*) are straightforward (see Supplementary Information). However, this relies on the fact that *z*_*t*_ ∈ {0, 1}. The observed covariates *x*_*t*_ follow no such restriction, which results in a transcendental equation for the *β*_*x*_ updates. In our implementation of the model, we solve this using an explicit BFGS optimization on each iteration. Moreover, we place non-hierarchical Gamma priors on these effects: λ_*xur*_ ∼ Gamma(*a*_*xur*_, *b*_*xur*_).

As stated above, for the latent states and baselines, we assume hierarchical priors. This allows us to model each neuron’s firing rate response to a particular stimulus as being drawn from a population response to that same stimulus. We also assume that the moment-to-moment noise in firing rates, *θ*_*m*_, follows a neuron-specific distribution. As a result of the form of this hierarchy given in Eq (3), the first piece in Eq (8) contains multiple terms of the form

$$\begin{array}{c} E_{q}[\sum_{u}\log p\left(\lambda_{u}|c,d\right)]=\sum_{u}E_{q}[\left(c-1\right)\log\lambda_{u}-cd\lambda_{u}+c\log cd-\log\Gamma \left(c\right)] \end{array}$$ (15)

In order to calculate the expectation, we make use of the following inequality [24]

$$\begin{array}{c} \sqrt{2\pi }\leq \frac{z!}{z^{z+\frac{1}{2}}e^{-z}}\leq e \end{array}$$ (16)

to lower bound the negative gamma function and approximate the above as

$$\begin{array}{c} \log p\left(\lambda \right)\geq \sum_{u}[\left(c-1\right)\left(\log\lambda_{u}+1\right)-cd\lambda_{u}+c\log d+\frac{1}{2}\log c] \end{array}$$ (17)

Clearly, the conditional probabilities for *c* and *d* are gamma in form, so that if we use priors *c* ∼ Gamma(*a*_*c*_, *b*_*c*_) and *d* ∼ Gamma(*a*_*d*_, *b*_*d*_) the posteriors have the form

$$c∼\text{Gamma}(a_{c}+\frac{U}{2},b_{c}+\sum_{u}E_{q}[d\lambda_{u}-\log\lambda_{u}-\log d-1])$$ (18)

$$d∼\text{Gamma}(a_{d}+UE_{q}\left[c\right],b_{d}+\sum_{u}E_{q}\left[c\lambda_{u}\right])$$ (19)

This basic form, with appropriate indices added, gives the update rules for the hyperparameter posteriors for λ_0_ and λ_*z*_. For *θ*, we simply set *c* = *s*_*u*_ and *d* = 1.

For each latent variable *z*, the Markov Chain parameters *π*_*k*_ and *A*_*k*_, together with the observation model Eq (11) determine a Hidden Markov Model, for which inference can be performed efficiently via conjugate updates and the well-known forward-backward algorithm [25]. More explicitly, given *π*, *A*, and the emission probabilities for the observations, log *p*(*N*|*z*), the forward-backward algorithm returns the probabilities *p*(*z*_*t*_ = *s*) (posterior marginal), *p*(*z*_*t*+1_ = *s*′, *z*_*t*_ = *s*) (two-slice marginal) and log *Z* (normalizing constant).

Our final algorithm is presented in Algorithm 1. Equation numbers reference posterior definitions in the text. Exact updates for the sufficient statistics are presented in Table 2 of S1 Text.

**Algorithm 1** Iterative update for variational inference

1: **procedure** Iterate

2:  Update baselines λ_0_                  ▷ conjugate Gamma Eq (12)

3:  Update baseline hyperparameters *γ*_0_       ▷ conjugate Gamma (Eqs 18 and 19)

4:  **for** *k* = 1 … *K* **do**

5:   Update firing rate effects λ_*zk*_              ▷ conjugate Gamma Eq (13)

6:   Update firing rate hyperparameters *γ*_*zk*_      ▷ conjugate Gamma (Eqs 18 and 19)

7:   Calculate expected log evidence *η*_*k*_                    ▷ (S13)

8:   Update Markov chain parameters ${\tilde{A}}_{k},{\tilde{\pi }}_{k}$              ▷ (S11, S12)

9:   *ξ*_*k*_, Ξ_*k*_, log *Z*_*k*_← FORWARD-BACKWARD ($\eta_{k},{\tilde{A}}_{k},{\tilde{\pi }}_{k}$)   ▷ [26, 27]

10:   **if** semi-Markov **then**

11:    Update duration distribution *p*_*k*_(*d*|*j*)         ▷ BFGS optimization (S25)

12:   **end if**

13:   Update cached *F*                           ▷ (S8)

14:  **end for**

15:  Update covariate firing effects λ_*x*_       ▷ BFGS optimization (Eq 14, S54, S55)

16:  Update cached *G*                            ▷ (S9)

17:  Update overdispersion *θ*            ▷ conjugate Gamma (Eqs 18 and 19)

18: **end procedure**

## Results

In this section, we report the results of three experiments illustrating the capabilities of our model. The first demonstrates the ability of our algorithm to recover ground truth latent features in a synthetic dataset with parameters similar to typical neural recording experiments. In the second and third, we use data from actual experiments in order to compare labels specified by experimenters with those recovered by our model. In each case, our model was only trained using stimulus identity, *not* experimenter labels, but nonetheless managed to recover key features that drove neural firing in the experiment. Code for all experiments and analysis is provided online (see Supplementary Information).

### Synthetic data

We generated synthetic data from the model for *U* = 100 neurons for *T* = 10,000 time bins of *dt* = 0.0333*s* (≈ 6min of movies at 30 frames per second). Assumed firing rates and effect sizes were realistic for cortical neurons, with mean baseline rates of 10 spikes/s and firing rate effects given by a Gamma(1, 1) distribution for *K*_data_ = 3 latent features. In addition, we included *R* = 3 known covariates generated according to Markov dynamics. For this experiment, we assumed that each unit was presented only once with the stimulus time series, so that *M*_*tu*_ = 1. That is, we tested a case in which inference was driven primarily by variability in population responses across stimuli rather than pooling of data across repetitions of the same stimulus. Moreover, to test the model’s ability to parsimoniously infer features, we set *K* = 5. That is, we asked the model to recover more features than were present in the data. Finally, we placed hierarchical priors on neurons’ baseline firing rates and sparse hierarchical priors on firing rate effects of latent states. We used 10 random restarts and iterated over parameter updates until the fractional change in L dropped below 10^−4^.

**Fig 3:**
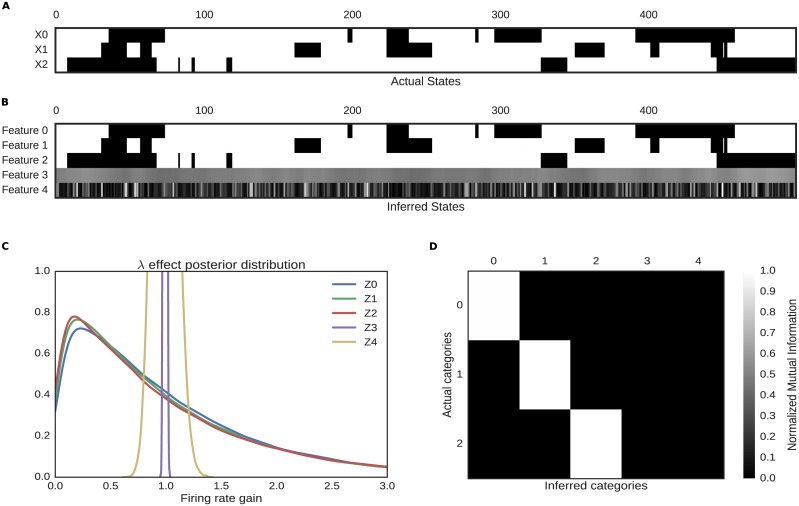
Comparison of actual and inferred states of the synthetic data. A: Ground truth binary latent features for a subset of stimulus times in the synthetic dataset. B: Recovered binary features for the same subset. Features have been reordered to facilitate comparisons with panel A. The unused features are in gray, indicating a high posterior uncertainty in the model. C: Population posterior distributions for inferred hyper parameters. Features 3 and 4 are effectively point masses around gain 1 (no population gain change in response to the feature), while features 1–3 approximate the Gamma(1, 1) data-generating model. D: Normalized mutual information between actual and inferred states.

As seen in Fig 3, the model correctly recovers only the features present in the original data. We quantified this by calculating the normalized mutual information $\hat{I}\equiv I\left(X,Y\right)/\sqrt{H(X)H(Y)}$, between the actual states and the inferred states, with *H*(*Z*) and *I* estimated by averaging the variational posteriors (both absolute and conditioned on observed states) across time. Note that superfluous features in the model have high posterior uncertainty for *z*_*k*_ and high posterior confidence for λ_*zk*_ around 1 (no effect).

### Labeled neural data

**Fig 4:**
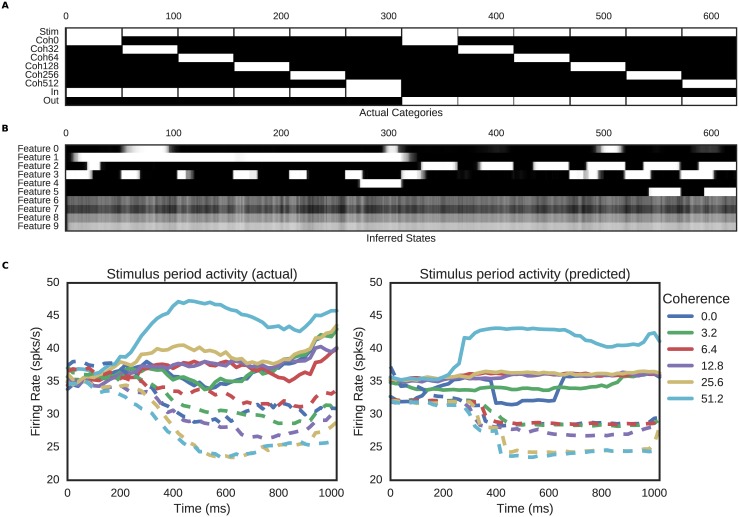
Comparison of actual and inferred states of the Roitman dataset. A: Experimental design features. Each vertical block represents a single type of trial (combination of stimulus coherence and choice location). Features present on a particular trial are plotted in white, and duration of each feature within the stimulus presentation period is indicated by the width of the bar in the horizontal direction. B: Recovered features from the model. Note that model features 6–9 are unused and that Features 1 & 2 closely track the In and Out features of the data, respectively. Shorter bars represent inferred features that lasted less than the full stimulus presentation period. C: Actual and predicted firing rates for the stimulus period. Note that the model infers stimulus categories from the data, including appropriate timing of differentiation between categories.

We applied our model to a well-studied neural data set comprising single neuron recordings from macaque area LIP collected during the performance of a perceptual discrimination task [28, 29]. In the experiment, stimuli consisted of randomly moving dots, some percentage of which moved coherently in either the preferred or anti-preferred direction of motion for each neuron. The animal’s task was to report the direction of motion. Thus, in addition to 5 coherence levels, each trial also varied based on whether the motion direction corresponded to the target in or out of the response field as depicted in Fig 4. (In the case of 0% coherence, the direction of motion was inherently ambiguous and coded according to the monkey’s eventual choice.) For our experiment, we only analyzed correct trials, on which the animal’s choice (target IN or OUT of response field) was synonymous with the direction of dot motion.

We fit a model with *K* = 10 features and *U* = 27 units to neural responses from the 1-second stimulus presentation period of the task. Spike counts corresponded to bins of *dt* = 20ms. For this experiment, units were individually recorded, so each unit experienced a different number of presentations of each stimulus condition, implying a ragged observation matrix. As a result, this dataset tests the model’s ability to leverage shared task structure across multiple sessions of recording, demonstrating that simultaneously recorded units are not required for inference of latent states.

Fig 4 shows the experimental labels from the concatenated stimulus periods, along with labels inferred by our model. Once again, the model has left some features unused, but correctly discerned differences between stimuli in the unlabeled data. Even more importantly, though given the opportunity to infer ten distinct stimulus classes, the model has made use of only five. Moreover, the discovered features clearly recapitulate the factorial design of the experiment, with the two most prominent features, *Z*_1_ and *Z*_2_, capturing complementary values of the variable with the largest effect in the experiment: whether or not the relevant target was inside our outside the receptive field of the recorded neuron. This difference can be observed in both the averaged experimental data and the predicted data from the model (see Fig 4C), where the largest differences are between the dotted and solid lines. Finally, we can ask whether the reconstructed firing rates are in quantitative agreement with the data estimates by calculating an RMS error for each curve in Fig 4C. That is, we calculate $\sqrt{\frac{E[{\left(f_{i}-f_{a}\right)}^{2}]}{E\left[f_{i}\right]E\left[f_{a}\right]}}$ for each unit, where *f*_*i*_ is the inferred firing rate from the model, *f*_*a*_ is the mean firing rate estimated from data, and expectations are taken across time bins. For our model, these values range from 4% to 12% across coherence levels.

But the model also reproduces less obvious features: it correctly discriminates between two identical stimulus conditions (0% coherence) based on the monkey’s eventual decision (In vs Out). In addition, the model correctly captures the initial 200ms “dead time” during the stimulus period, in which firing rates remain at pre-stimulus baseline. (Note that the timing is locked to the stimulus and consistent across trials, not idiosyncratic to each trial as in [30].) Finally, the model resists detection of features with little support in the experimental data. For instance, while feature *Z*_4_ captures the large difference between 50% coherence and other stimuli, the model does not infer a difference between intermediate coherence levels that are indistinguishable in this particular dataset. That is, mismatches between ground truth labels and model-inferred features here reflect underlying ambiguities in the neural data, while the model’s inferred features correctly pick out those combinations of variables most responsible for differences in spiking across conditions.

### Visual category data

**Fig 5:**
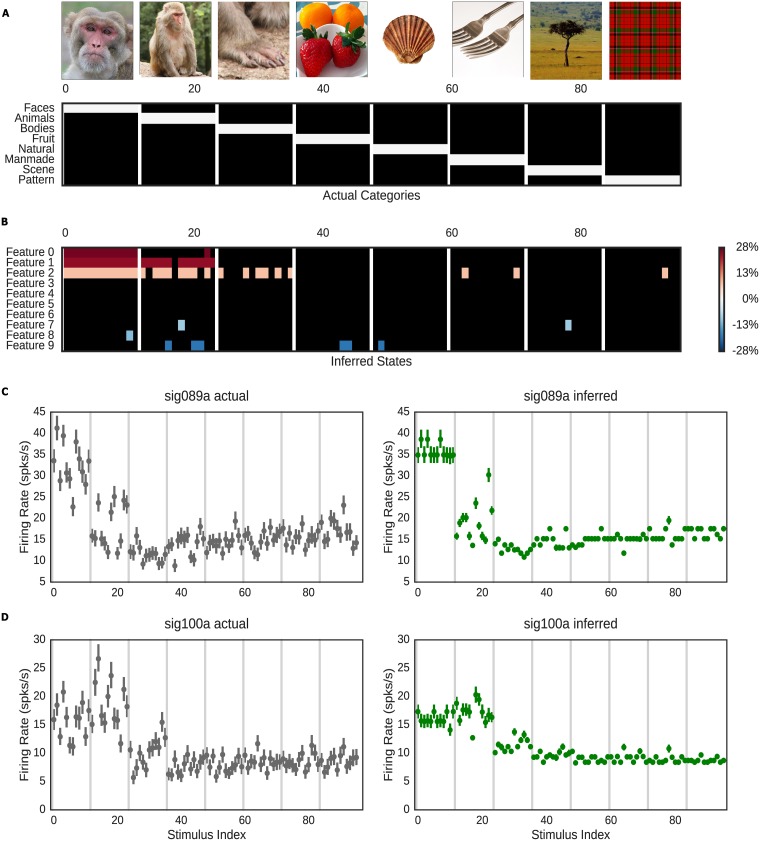
Comparison of actual and inferred states of the macaque dataset. A: Experimenter-determined features for the IT data set. 96 stimuli comprising 8 categories were presented in 1483 trials, with each stimulus presented to each neuron ∼15 times. B: The inferred states from our model. Color represents the mean percent change in firing rate across the population in response to each feature. For clarity, features with mean population effects <5% are not plotted. The model has inferred features corresponding to monkey close-ups, whole monkey photos, and most close-ups of monkey body parts. C: Actual and predicted spikes per second across all stimulus of neuron 089a. D. Actual and predicted spikes per second across all stimulus of neuron 100a. Error bars for data represent 95% credible intervals for firing rates inferred from observed data using a Poisson model with weak priors. Error bars on predictions are 95% credible intervals based on simulation from the approximate posterior for the plotted unit. Images copyright Geoff Gallice, kimubert/Flickr, dvs/Flickr, Julien Harneis, and Celtus/WikiMedia under CC-BY. Second and third monkey images copyright J.M. Garg (used with permission).

As a second test of our model, we applied our algorithm to a designed structured stimuli dataset comprising *U* = 56 neurons from macaque inferotemporal cortex [31]. These neurons were repeatedly presented with 96 stimuli comprising 8 categories (*M* = 1483 total trials, with each stimulus exposed between 12 to 19 times to each unit) comprising monkey faces, monkey bodies, whole monkeys, natural scenes, food, manmade objects, and patterns (Fig 5A). Data consisted of spike time series, which we binned into a 300ms pre-stimulus baseline, a 300ms stimulus presentation period, and a 300ms post-stimulus period. Three trials were excluded because of the abnormal stimulus presentation period. To maximize interpretability of the results, we placed strong priors on the *π*_*k*_ to formalize the assumption that all features were off during the baseline period. We also modeled overdispersion with extremely weak priors to encourage the model to attribute fluctuations in firing to noise in preference to feature detection. We again fit *K* = 10 features with sparse hierarchical priors on population responses.

**Fig 6:**
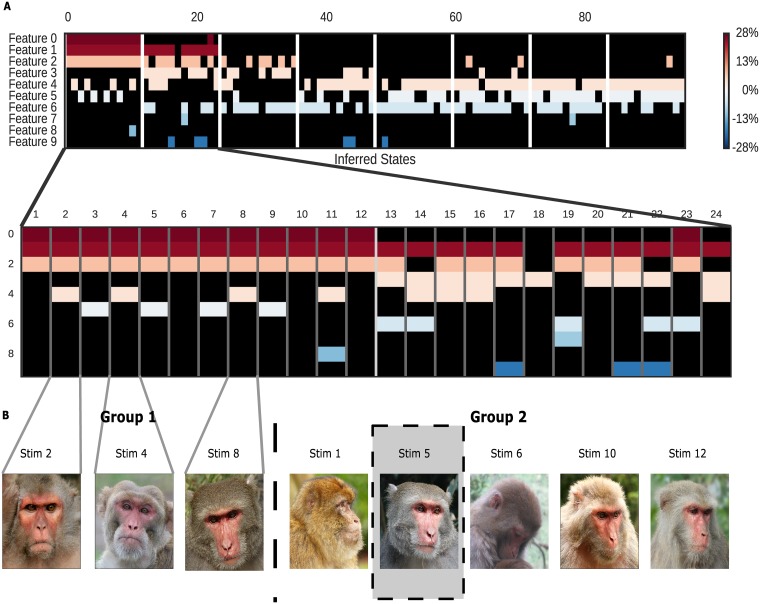
Small features suggest additional neural hypotheses. A: Zoomed-in view of Fig 5A, focusing on the first 24 images. B: The feature combinations 0&1&2&4 (Group 1) and 0&1&2 (Group 2) are distinguished by direct vs. indirect gaze. Only Stimulus 5, coded 0&1&2&5, is missing from Group 2. Images are for illustration only. Stims 2, 4, and 10 correspond to images in the original data set; other images approximate stimuli for which publication permission could not be obtained. Images copyright jinterwas/Flickr (Stim 2), Geoff Gallice (Stim 4) under CC-BY. Stim 10 copyright J.M. Garg (used with permission). All others in the public domain.

The inferred categories based on binned population responses are shown in Fig 5B. For clarity, in Fig 5, we only show population mean effects with a > 5% gain modulation sorted from the highest to the lowest, though the full set of inferred states can be found in Fig 6. Out of the original categories, our model successfully recovers three features clearly corresponding to categories involving monkeys (Features 0–2). These can be viewed additively, with Feature 0 exclusive to monkey face close-ups, Feature 1 any photo containing a monkey face, either near or far; and Feature 2 any image containing a monkey body part (including faces); but as we will argue, given the nature of the model, it may be better to view these as a “combinatorial” code, with monkey close-ups encoded as 0&1&2 (∼ 59.46% increase in firing), whole monkeys as 1&2 (∼ 32.47% increase), and monkey body parts as 2 (∼ 7.62% increase). Of course, this is consistent with what was found in [31], though our model used no labels on the images. And our interpretation that these neurons are sensitive to close-ups and faraway face and body parts is consistent with findings by another study using different experimental settings [32].

Again, as noted above, our results in Fig 5A and 5B indicate predicted population responses, derived from the hierarchical prior. As evidenced in Fig 5C and 5D, individual neuron effects could be much larger. These panels show data for two example units, along with the model’s prediction. Clearly, the model recapitulates the largest distinctions between images in the data, though the assumption that firing rates should be the same for all images with similar features fails to capture some variability in the results. Here, RMS errors range from 16% to 238% across units, with most units showing at least qualitative agreement from only a handful of presentations of each stimulus. Even so, uncertainties in the predicted firing rates are also in line with uncertainties from those of observed rates, indicating that our model is correctly accounting for trial-to-trial noise.

Finally, even the weaker, sparser features inferred by our model captured intriguing additional information. As shown in Fig 6, Feature 4, a feature only weakly present in the population as a whole (and thus ignored in Fig 6A), when combined with the stronger Features 0, 1, and 2, successfully distinguishes between the monkey close-ups with direct and averted gaze. (Stimulus 5, with averted gaze, is additionally tagged with Feature 5, which we view as an imperfect match.) Thus, despite the fact that Feature 4 is barely a 3.4% gain change over the population, it suggests a link between neural firing and gaze direction, one for which there happens to be ample evidence [33, 34]. Similarly, Feature 5, barely a 1.1% effect, correctly tags three of the four close-ups with rightward gaze (with one false positive). Clearly, neither of these results is dispositive in this particular dataset, but in the absence of hypotheses about the effect of head orientation and gaze on neuronal firing, these minor features might suggest hypotheses for future experiments.

An additional feature of our approach is that the generated labels provide a concise and fairly complete summary of the stimulus-related activity of all neural recordings, which can be observed by comparing the categorization performance of decoded neural activity to the categorization performance of the decoded features. Although our model is not a data compression method, it nonetheless preserves most of the information about image category contained in the *N* = 56 dimensional spike counts via a 10-dimensional binary code. That is, using a sparse logistic regression on two-bit and three-bit combinations of our features to predict stimulus category ties and outperforms, respectively a multinomial logistic regression on the raw spike counts (see Supplementary Information).

## Discussion

Here, we have proposed and implemented a method for learning features in stimuli via the responses of populations of spiking neurons. This work addresses a growing trend in systems neuroscience—the increasing use of rich and unstructured or structured stimulus sets—without requiring either expert labeling or a metric on the stimulus space. As such, we expect it to be of particular use in disciplines like social neuroscience, olfaction, and other areas in which the real world is complex and strong hypotheses about the forms of the neural code are lacking. By learning features of interest to neural populations directly from neural data, we stand to generate unexpected, more accurate (less biased) hypotheses regarding the neural representation of the external world.

Here, we have validated this method using structured, labeled stimuli more typical of neuroscience experiments, showing that our model is capable of parsimoniously and correctly inferring features in the low signal-to-noise regime of cortical activity, even in the case of independently recorded neurons. Furthermore, by employing a fully variational, Bayesian approach to inference, we gain three key advantages: First, we gain the advantages of Bayesianism in general: estimates of confidence in inferences, parsimony and regularization via priors, and the ability to do principled model comparison. Second, variational methods scale well to large datasets and can be easily parallelized when combining data from multiple recording sessions. Finally, variational methods are fast, in that they typically converge within only a few tens of iterations and in many case (such as ours) can be implemented using explicit coordinate update rules, eliminating the need to tune a learning rate.

Finally, even small features in our model recapitulated known physiological results regarding face encoding in single neurons. And while these features alone might not provide proof positive of, e.g., viewpoint tuning, similar findings would be valuable in generating hypotheses in cases where the stimulus space and its neural correlates remain poorly understood. Thus our model facilitates an iterative experimental process: subjects are first be exposed to large, heterogeneous data; stimuli are then tagged based on neural responses; and finally, features with the largest effects are used to refine the set until it most accurately represents those stimuli with the largest neural correlates. Combined with the modularity of this and similar approaches, such models provide a promising opportunity to “build out” additional features that will meet the challenges of the next generation of experimental data.

## Supporting information

S1 Text Mathematical details. Derivation of ELBO and Inference.
(PDF)

S2 Text Inferred latents as classification features. (PDF)

S3 Text Effects of bin size and dynamics. (PDF)
