# Supplementary material for: Neuron’s eye view: Inferring features of complex stimuli from neural responses
Source: PLoS Comput Biol. 2017 Aug 21;13(8):e1005645. doi: 10.1371/journal.pcbi.1005645 (PMC5578681; doi:10.1371/journal.pcbi.1005645)
Supplement: S1 Text — Derivation of ELBO and Inference. (PDF) [file pcbi.1005645.s001.pdf]

S1 Text. Mathematical details
Derivation of ELBO and Inference

1 Evidence Lower Bound (ELBO)

Here we derive the evidence lower bound (ELBO) used as a variational objective by our inference algorithm. That is, we want to calculate

$$\mathcal{L} \equiv \mathbb{E}_q \left[\log \frac{p(\Theta|N)}{q(\Theta)} \right] = \mathbb{E}_q \left[\log p(\Theta|N) \right] + \mathcal{H}[q(\Theta)] \tag{1}$$

From [1], this can be written

$$\mathcal{L} = \mathbb{E}_{q(\pi)} \left[\log \frac{p(\pi)}{q(\pi)} \right] + \mathbb{E}_{q(A)} \left[\log \frac{p(A)}{q(A)} \right] + \mathbb{E}_q \left[\log \frac{p(N, z|\lambda, A, \pi)}{q(z)} \right] + \mathbb{E}_{q(\theta)} \left[\log \frac{p(\theta)}{q(\theta)} \right] + \mathbb{E}_{q(\lambda)} \left[\log \frac{p(\lambda)}{q(\lambda)} \right] + \mathbb{E}_{q(\gamma)} \left[\log \frac{p(\gamma)}{q(\gamma)} \right] \tag{2}$$

For the first two terms, updates are standard and covered in [1]. The rest we do piece-by-piece below:

1.1 Log evidence

We would like to calculate $\mathbb{E}_q \left[\log \frac{p(N,z|x,\Theta)}{q(z)} \right]$. To do this, we make use of expectations calculated via the posteriors returned from the forward-backward algorithm

$$\xi_t \equiv p(z_t|N, \theta) \qquad \Xi_{t,ij} \equiv p(z_{t+1} = j, z_t = i|N, \theta) \qquad \log Z_t = \log p(N_{t+1}|N_t, \Theta) \tag{3}$$

Here, we have suppressed the latent feature index k and abuse notation by writing the observation index as t, but in the case of multiple observations at a given time, we pool across units and presentations: $N_t \equiv \sum_{m;t(m)=t} N_m$. From this, we can write

$$\mathbb{E}_q \left[\log p(N, z|x, \Theta) \right] = \sum_{mkr} \left[N_m \left(\overline{\log \theta_m} + \overline{\log \lambda_{0u(m)}} + \xi_{t(m)k} \overline{\log \lambda_{zuk}} + x_{t(m)r} \overline{\log \lambda_{xu(m)r}} \right) \right] - \sum_m \overline{\theta_m} \mathbb{E}_q \left[\Lambda_{t(m)u(m)} \right] + \sum_{tk} \left[\mathrm{tr} \left(\Xi_{tk} \overline{\log A_k^T} \right) + \xi_{0k}^T \overline{\log \pi_k} \right] + \text{constant} \tag{4}$$

where for notational simplicity, we have replaced expectations with overlines: $\overline{x} \equiv \mathbb{E}[x]$. In what follows, we will drop the irrelevant constant. For $\overline{\log y}$, where $y \in \{\theta, \lambda_0, \lambda_z, \lambda_x\}$, the assumption $q(y) = \mathrm{Ga}(\alpha, \beta)$ gives

$$\overline{\log y} = \psi(\alpha) - \log \beta \tag{5}$$

with $\psi(x)$ the digamma function. Likewise, the expectation $\overline{\theta}$ is straightforward. For the expectation of the rate, we have

$$\mathbb{E}_q \left[\lambda_{0u} \prod_k (\lambda_{zuk})^{z_{tk}} \prod_r (\lambda_{xur})^{x_{tr}} \right] = \frac{\alpha_{0u}}{\beta_{0u}} \prod_k \left(1 - \xi_{tk} + \xi_{tk} \frac{\alpha_{zuk}}{\beta_{zuk}} \right) \prod_r \frac{1}{\beta_{xur}^{x_{tr}}} \frac{\Gamma(\alpha_{xur} + x_{tr})}{\Gamma(\alpha_{xur})} \tag{6}$$

However, for $\alpha \gg x$, we have $\Gamma(\alpha + x)/\Gamma(\alpha) \approx \alpha^x$, so that we can write

$$\mathbb{E}_q[\Lambda_{tu}] = H_{0u} F_{tu} G_{tu} \tag{7}$$

with $G_{tu} \approx \prod_r (\alpha_{xur}/\beta_{xur})^{x_{tr}}$. In addition, it will later be useful to have the expectation over *all except* a particular feature k or r, for which we define

$$F_{tuk} \equiv \prod_{k' \neq k} \left(1 - \xi_{tk'} + \xi_{tk'} \frac{\alpha_{zuk'}}{\beta_{zuk'}}\right) \tag{8}$$

$$G_{tur} \equiv \prod_{r' \neq r} \left(\frac{\alpha_{xur'}}{\beta_{xur'}}\right)^{x_{tr'}} \tag{9}$$

Finally, we want the entropy of the variational posterior over z, $\mathbb{E}_q[-\log q(z)]$. We can write this in the form

$$-\sum_{tk} \left[\xi_{tk}^T \eta_{tk} + \operatorname{tr}\left(\Xi_{tk} \tilde{A}_k^T\right) + \xi_{0k}^T \tilde{\pi}_k - \log Z_{tk}\right] \tag{10}$$

with $(\eta, \tilde{A}, \tilde{\pi})$ the parameters of the variational posterior corresponding to the emission, transition, and initial state probabilities of the Markov chain (interpreted as matrices) and Z the normalization constant. From [1], we have that variational updates should give

$$\tilde{A}_k \leftarrow \overline{\log A_k} \tag{11}$$

$$\tilde{\pi}_k \leftarrow \overline{\log \pi_k} \tag{12}$$

while the effective emission probabilities in the "on" $(z = 1)$ state of the HMM are

$$\eta_{tk} \leftarrow \delta_{z_{tk},1} \sum_{m;t(m)=t} N_m \overline{\log \lambda_{zu(m)k}} - \sum_{m;t(m)=t} \overline{\theta_m} H_{0u(m)} F_{tku(m)} G_{tu(m)} \tag{13}$$

Given these update rules, we can then alternate between calculating $(\eta, \tilde{A}, \tilde{\pi})$, performing forward-backward to get $(\xi, \Xi, \log Z)$ and recalculating $(\eta, \tilde{A}, \tilde{\pi})$.

1.2 Overdispersion, firing rate effects

Both the case of $p(\theta)$ and $p(\lambda)$ are straightforward. If we ignore subscripts and write $p(y) = \mathrm{Ga}(a, b)$, $q(y) = \mathrm{Ga}(\alpha, \beta)$, then

$$\mathbb{E}_q\left[\log \frac{p(y)}{q(y)}\right] = (\overline{a} - 1)\overline{\log y} + \overline{b}\overline{y} + \mathcal{H}[q(y)] \tag{14}$$

where again, $\overline{\log y}$, $\overline{y}$ and $\mathcal{H}[q(y)]$ are straightforward properties of the Gamma distribution. Expectations of the prior parameters are listed in Table 1. Note in the last line that there is no expectation, since we have not assumed a hierarchy over firing rate effects for the covariates, x.

1.3 Hyperparameters

As shown in the main text, the hyperparameters c and d, given gamma priors, have conjugate gamma posteriors, so that their contribution to the evidence lower bound, $\mathbb{E}_q\left[\log \frac{p(\gamma)}{q(\gamma)}\right]$ is a sum of terms of the form

$$(a_c - 1)\overline{\log c} + b_c \overline{c} + \mathcal{H}[q(c)] + (a_d - 1)\overline{\log d} + b_d \overline{d} + \mathcal{H}[q(d)] \tag{15}$$

Table 1. Expectations of prior parameters for overdispersion and firing rates

**Variable**	$\mathbb{E}_q[a]$	$\mathbb{E}_q[b]$
θ	$\overline{s}$	$\overline{s}$
λ_0	$\overline{c_0}$	$\overline{c_0 d_0}$
λ_z	$\overline{c_z}$	$\overline{c_z d_z}$
λ_x	a_x	b_x

In other words, these are straightforward gamma expectations, functions of the prior parameters a and b for each variable and the corresponding posterior parameters α and β. Similarly, the overdispersion terms are exactly the same with the substitutions $c \to s$, $d \to 1$.

As we will see below, the expectations under the variational posterior of s, c, and d are themselves straightforward to calculate.

1.4 Autocorrelated noise

In the main text, we assumed the θ_m to be uncorrelated. However, it is possible to model temporal autocorrelation among the θ_m when observations correspond to the same neuron at successive time points. More specifically, let us replace the observation index m by τ, the experimental clock time. We then write a particular observation as corresponding to a stimulus time $t(\tau)$ and a set of units $u(\tau)$, which, for simplicity, we will assume fixed and simply write as u.[1] To model the autocorrelation of noise across successive times, we then write

$$N_m \sim \mathrm{Pois}(\Lambda_{t(\tau),u}\theta_{\tau u}) \qquad \text{Assuming } \theta_{\tau u} = \phi_{\tau u}\theta_{\tau-1,u} \tag{16}$$

If $\phi_{0u} = \theta_{0u}$, we then have $\theta_{\tau u} = \prod_{\tau' \le \tau} \phi_{\tau' u}$. In essence, this is a log-autoregressive process in which the innovations are not necessarily normally distributed. In fact, if we further assume that $p(\phi_{\tau u}) = \mathrm{Ga}(s_u, r_u)$ and $q(\phi_{\tau u}) = \mathrm{Ga}(\omega_{\tau u}, \zeta_{\tau u})$, we can once again make use of conjugate updates.

Given these assumptions, we need to make the following modifications for the third and fifth terms in the evidence lower bound of Eq (4):

$$\sum_{mkr} N_m \xi_{t(m)k} \overline{\log \lambda_{zuk}} \to \sum_{mkr} N_m \xi_{t(m)k} \overline{\log \lambda_{zuk}} + \sum_{\tau u} \overline{\log \phi_{\tau u}} \sum_{\tau' \ge \tau} N_{\tau' u} \tag{17}$$

$$-\sum_m \overline{\theta_m} \mathbb{E}_q\left[\Lambda_{t(m)u(m)}\right] \to -\sum_{\tau u} H_{0u} F_{\tau k u} G_{\tau u} \prod_{\tau' \le \tau} \frac{\omega_{\tau' u}}{\zeta_{\tau' u}} \tag{18}$$

Thus the effective emission probabilities in State 1 now become:

$$\eta_{tk} \to \delta_{z_{tk},1} \sum_m N_m \overline{\log \lambda_{zuk}} - \sum_{u; t(\tau)=t} H_{0u} F_{\tau k u} G_{\tau u} \prod_{\tau' \le \tau} \frac{\omega_{\tau' u}}{\zeta_{\tau' u}} \tag{19}$$

1.5 Latent states, semi-Markov dynamics

We model each of the latent states z_{tk} as an independent Markov process for each feature k. That is, each k indexes an independent Markov chain with initial state probability $\pi_k \sim \mathrm{Dir}(\alpha_\pi)$ and transition matrix $A_k \sim \mathrm{Dir}(\alpha_A)$. For the semi-Markov case, we assume that the dwell times in each state are distributed independently for

[1] The generalization to partially overlapping neurons and stimuli is straightforward but complex and notationally cumbersome.

each chain according to an integer-valued, truncated lognormal distribution with support on the integers $1 \dots D$:

$$p_k(d|z=j) = \text{Log-Normal}(d|m_{jk}, s_{jk}^2)/W_{jk} \tag{20}$$

$$W_{jk} = \sum_{d=1}^{D} \text{Log-Normal}(d|m_{jk}, s_{jk}^2) \tag{21}$$

Note that we have allowed the dwell time distribution to depend on both the feature k and the state of the Markov chain j. In addition, we put independent Normal-Gamma priors on the mean (m_{kj}) and precision ($\tau_{kj} \equiv s_{kj}^{-2}$) parameters of the distribution: $(m, \tau) \sim \text{NG}(\mu, \lambda, \alpha, \beta)$.

In this case, we additionally need to perform inference on the parameters (m, τ) of the dwell time distributions for each state. In the case of continuous dwell times, our model in Equation 20 would have $W = 1$ and be conjugate to the Normal-Gamma prior on (m, τ), but the restriction to discrete dwell times requires us to again lower bound the variational objective:

$$\mathbb{E}_q\left[-\log W_{jk}\right] = \mathbb{E}_q\left[-\log\left(\sum_{d=1}^{D} p(d|j)\right)\right] \geq -\log \sum_{d=1}^{D} \mathbb{E}_q\left[p(d|j)\right] \tag{22}$$

This correction for truncation must then be added to $\mathbb{E}_q[p(z|\Theta)]$. For inference in the semi-Markov case, we use an extension of the forward-backward algorithm [2], as well as the algorithms in [3, 4], at the expense of computational complexity $\mathcal{O}(SDT)$ $(S = 2)$ per latent state, to calculate $q(z_k)$. For the $4SK$ hyperparameters of the Normal-Gamma distribution, we perform an explicit BFGS optimization on the $4S$ parameters of each chain on each iteration (detailed in Subsection 2.3).

The new ELBO for the semi-Markov model thus adds to the terms involving Ξ and ξ_0 in (4) an additional piece:

$$\sum_{djk} C(d,j,k) \mathbb{E}_q\left[\log p_k(d|j) - \log\left(\sum_{d=1}^{D} p_k(d|j)\right)\right] \geq$$
$$\sum_{djk} C(d,j,k)\left[\mathbb{E}_q\left[\log p_k(d|j)\right] - \log \sum_{d=1}^{D} \mathbb{E}_q\left[p_k(d|j)\right]\right] \tag{23}$$

where as noted above, the second term inside the expectation arises from the need to normalize $p(d|j)$ over discrete times and the inequality follows from Jensen's Inequality. Here, d is the duration variable, j labels states of the chain (here 0 and 1), and k, as elsewhere, labels chains [2–4]. C is defined for each state and chain as the probability of a dwell time d in state j *conditioned on* the event of just having transitioned to j[2] [3]

Thus the objective to be optimized is

$$\sum_{djk} C(d,j,k)\left[\mathbb{E}_q\left[\log p_k(d|j)\right] - \log \sum_{d=1}^{D} \mathbb{E}_q\left[p_k(d|j)\right]\right] + \mathbb{E}_q\left[\log \frac{p(m,\tau)}{q(m,\tau)}\right] \tag{25}$$

[2]Thus C is equivalent to $\mathcal{D}_{t|T}$ in [2].

[3] We also note that, just as the emission, transition, and initial state probabilities have their counterparts in variational parameters $(\eta, \tilde{A}, \tilde{\pi})$ in $q(z)$, so C is matched by a term $-\sum_{djk} C(d,j,k)\nu_{djk}$ in $\mathbb{E}_q[-\log q]$. And in analogy with the HMM case, variation with respect to ν gives

$$\nu_{djk} = \mathbb{E}_q\left[\log p_k(d|j)\right] - \log \sum_{d=1}^{D} \mathbb{E}_q\left[p_k(d|j)\right], \tag{24}$$

implying that there are cancellations between $\log p(N, z|\Theta)$ and $\log q(z)$ in $\mathcal{L}$ and care must be taken when calculating it [1].

We focus on each of these and their updates below.

1.5.1 Contributions to the ELBO

For the case of $p(d|m, s^2)$ Log-Normal and $(m, \tau = s^{-2})$ Normal-Gamma, the terms $\mathbb{E}_q[\log p(d|j)]$ involve only routine expectations of natural parameters of the Normal-Gamma, and similarly for the expected log prior and entropy in the last term. Writing the delay distribution in the exponential family form with base measure h and natural parameter η

$$p(d|z = i) = h(d) \exp(\eta_i \cdot T(d) - A(\eta_i)) \tag{26}$$

yields for the lognormal distribution $\log d \sim \mathcal{N}(m, s^2)$

$$h(d) = \frac{1}{\sqrt{2\pi d}} \tag{27}$$

$$T(d) = \begin{bmatrix} \log d \\ (\log d)^2 \end{bmatrix} \tag{28}$$

$$\eta = \begin{bmatrix} \frac{m}{s^2} \\ -\frac{1}{2s^2} \end{bmatrix} \tag{29}$$

$$A(\eta) = \frac{m^2}{2s^2} + \log s \tag{30}$$

If we then put a Normal-Gamma posterior on (m, τ) with parameters $(\mu, \lambda, \alpha, \beta)$, we can easily calculate

$$\mathbb{E}_q[\eta] = \mathbb{E}_q \begin{bmatrix} m\tau \\ -\frac{1}{2}\tau \end{bmatrix} = \begin{bmatrix} \mu\frac{\alpha}{\beta} \\ -\frac{\alpha}{2\beta} \end{bmatrix} \tag{31}$$

$$\mathbb{E}_q[A(\eta)] = \mathbb{E}_q \left[\frac{1}{2}m^2\tau - \frac{1}{2}\log\tau\right] \tag{32}$$

$$= \frac{1}{2}\mathbb{E}_\tau \left[\left(\mu^2 + \frac{1}{\lambda\tau}\right)\tau\right] - \frac{1}{2}(\psi(\alpha) - \log\beta) \tag{33}$$

$$= \frac{1}{2}\left(\mu^2\frac{\alpha}{\beta} + \frac{1}{\lambda}\right) - \frac{1}{2}(\psi(\alpha) - \log\beta) \tag{34}$$

The relevant piece of the evidence lower bound for updating $(\mu, \lambda, \alpha, \beta)$ is

$$\sum_{i,d} C(d, i)[\log h(d) + \mathbb{E}_q[\eta_i] \cdot T(d) - \mathbb{E}_q[A(\eta_i)] + \mathbb{E}_q \left[\log \frac{p(m, \tau)}{q(m, \tau)}\right] \tag{35}$$

again, with $q(m, \tau) = \text{Normal-Gamma}(\mu, \lambda, \alpha, \beta)$. We can thus write

$$\begin{aligned}\mathbb{E}_q[\log p(d|j)] &= -\frac{1}{2}\log 2\pi C_0 - C_1 + \mathbb{E}_q[m\tau]C_1 - \frac{1}{2}\mathbb{E}_q[\tau]C_2 \\ &\quad - \frac{1}{2}C_0\mathbb{E}_q[m^2\tau] + \frac{1}{2}C_0\mathbb{E}_q[\log\tau] \\ &= G + F \cdot T(\alpha, \beta, \lambda, \mu)\end{aligned} \tag{36}$$

where

$$G \equiv -\frac{1}{2}\log 2\pi C_0 - C_1 \tag{37}$$

$$F \cdot T = \begin{bmatrix} \frac{C_0}{2} & -\frac{C_2}{2} & C_1 & -\frac{C_0}{2} \end{bmatrix} \cdot \mathbb{E}_q \begin{bmatrix} \log\tau \\ \tau \\ m\tau \\ m^2\tau \end{bmatrix} \tag{38}$$

$$C_0 \equiv \sum_{id} C_{id} \tag{39}$$

$$C_1 \equiv \sum_{id} C_{id}\log d \tag{40}$$

$$C_2 \equiv \sum_{id} C_{id}(\log d)^2 \tag{41}$$

For calculating the final term in (25), we can make use of conjugacy (since $p(m,\tau|\alpha_0,\beta_0,\lambda_0,\mu_0)$ is itself Normal-Gamma) and the fact that (36) only depends linearly on the expected sufficient statistics of q to write the contribution of $\mathbb{E}_q[\log p(m,\tau)]$ as an update to F:

$$F' = \begin{bmatrix} \frac{C_0}{2} + \alpha_0 \\ -\frac{C_2}{2} - \beta_0 - \frac{\lambda_0\mu_0^2}{2} \\ C_1 + \lambda_0\mu_0 \\ -\frac{C_0}{2} - \frac{\lambda_0}{2} \end{bmatrix} \tag{42}$$

Moreover, we can make use of the standard entropy formula for the Normal-Gamma distribution:

$$\begin{aligned} H[q] &= \mathbb{E}_q[-\log q] = \mathbb{E}_q[-\log q(\tau) - \log q(m|\tau)] \\ &= H_g(\alpha,\beta) + \mathbb{E}_q\left[-\log\sqrt{\frac{\lambda\tau}{2\pi}} + \frac{\lambda\tau}{2}(m-\mu)^2\right] \\ &= \alpha - \log\beta + \log\Gamma(\alpha) + (1-\alpha)\psi(\alpha) \\ &- \frac{1}{2}\log\frac{\lambda}{2\pi} - \frac{1}{2}\left(\psi(\alpha) - \log\beta\right) + \frac{1}{2} \end{aligned} \tag{43}$$

Only slightly more complicated is the term arising from the normalization constant, which in the standard $(\mu,\lambda,\alpha,\beta)$ parameterization of the Normal-Gamma takes the form

$$\mathbb{E}_q[p(d|m,\tau)] = \int d\tau dm\; p(d|i,m,\tau)q(m,\tau) = \frac{1}{\sqrt{2\pi}d}\sqrt{\frac{\lambda}{1+\lambda}}\frac{\Gamma(\alpha+1/2)}{\Gamma(\alpha)}\frac{\beta^{-\frac{1}{2}}}{\hat{\beta}^{\alpha+\frac{1}{2}}} \tag{44}$$

with

$$\hat{\beta} \equiv 1 + \frac{1}{2\beta}\frac{\lambda}{1+\lambda}(\log d - \mu)^2 \tag{45}$$

This can be derived either by performing the integral directly or by noting that the result should be proportional to the posterior (Normal-Gamma, by conjugacy) of (m,τ) after having observed a single data point, $\log d$.

Taken together, (36) (with $F \to F'$), (43), and (44) allow (25) to be calculated directly as a function of the variational parameters $(\alpha,\beta,\lambda,\mu)$ of $q(m,\tau)$. Were it not for the normalization constant arising from the restriction of discrete d, the updates resulting from optimizing this objective would reduce by conjugacy to simple paramater updates. With that additional term, we are forced to use a brute-force BFGS optimization step instead (see 2.3 below).

1.5.2 Forward-Backward Algorithm for HSMM: Notation

Here, we follow Yu and Kobayashi, mutatis mutandis. Define:

$$\begin{aligned}
\psi_t(m) &= p(y_t|z_t = m) && \text{observation probability} \\
\alpha_{t|x}(m,d) &= p(z_t = m, d_t = d|y_{1:x}) && \text{condition on data} \\
\gamma_{t|x}(m) &= \sum_d \alpha_{t|x}(m,d) && \text{marginalize out } d \\
\psi_t^*(m) &= \frac{\alpha_{t|t}(m,d)}{\alpha_{t|t-1}(m,d)} = \frac{\psi_t(m)}{p(y_t|y_{1:t-1})} \\
r_t^{-1} &= p(y_t|y_{1:t-1}) = \sum_{m,d} \alpha_{t|t-1}(m,d)\psi_t(m) \\
&= \sum_m \gamma_{t|t-1}(m)\psi_t(m) \\
Z &= p(y_{1:T}) = \left(\prod_{t=1}^T r_t\right)^{-1} && \text{probability of data} \\
\mathcal{D}_{t|x}(m,d) &= p(z_t = m, d_t = d, d_{t-1} = 1|y_{1:x}) && \to (m,d) \\
\mathcal{T}_{t|x}(n,m) &= p(z_t = n, z_{t-1} = m, d_{t-1} = 1|y_{1:x}) && m \to n \\
\mathcal{E}_t(m) &= p(z_t = m, d_t = 1|y_{1:t}) = \alpha_{t|t-1}(m,1)\psi_t^*(m) && m \text{ ends at } t \\
\mathcal{S}_t(m) &= p(d_t = 1, z_{t+1} = m|y_{1:t}) = \sum_n A(m,n)\mathcal{E}_t(n) && m \text{ starts at } t+1
\end{aligned}$$

and for the backward pieces

$$\begin{aligned}
\beta_t(m,d) &= \frac{p(z_t = m, d_t = d|y_{1:T})}{p(z_t = m, d_t = d|y_{1:t-1})} = \frac{p(y_{t:T}|z_t = m, d_t = d)}{p(y_{t:T}|y_{1:t-1})} \\
\mathcal{E}_t^*(m) &= \frac{p(y_{t:T}|z_t = m, d_{t-1} = 1)}{p(y_{t:T}|y_{1:t-1})} = \sum_d p_m(d)\beta_t(m,d) \\
\mathcal{S}_t^*(m) &= \frac{p(y_{t:T}|z_{t-1} = m, d_{t-1} = 1)}{p(y_{t:T}|y_{1:t-1})} = \sum_n \mathcal{E}_t^*(n)A(n,m)
\end{aligned}$$

These quantities then allow us to write the posterior probabilities

$$\begin{aligned}
\alpha_{t|T}(m,d) &= \beta_t(m,d)\alpha_{t|t-1}(m,d) && \text{posterior prob of } (m,d) \\
\mathcal{T}_{t|T}(n,m) &= \mathcal{E}_t^*(n)A(n,m)\mathcal{E}_{t-1}(m) && \text{posterior prob of } m \to n \\
\mathcal{D}_{t|T}(m,d) &= \beta_t(m,d)p_m(d)\mathcal{S}_{t-1}(m) && \text{posterior prob of entering } (m,d) \text{ at } t
\end{aligned}$$

1.5.3 Forward

From Yu and Kobayashi, we have the following forward pass:

1. $\alpha_{1|0}(m,d) = \pi_m p_m(d)$ for time 1; otherwise, use

$$\alpha_{t|t-1}(m,d) = S_{t-1}(m)p_m(d) + \psi_{t-1}^*(m)\alpha_{t-1|t-2}(m,d+1) \tag{46}$$

2. Use $\alpha_{t|t-1}$ and ψ_t to get r_t; together, these give ψ_t^*. Store r_t
3. Calculate and save $\mathcal{E}_t$.
4. Calculate $\mathcal{S}_t$.
5. Loop.

1.5.4 Backward

Again following Yu and Kobayashi:

1. Initialize $\beta_T(m,d) = \psi^*_T(m) = \psi_T(m) r_T$
2. $\psi^*_t(m) = \psi_t(m) r_t$
3. Calculate $\beta_t(m,d)$ by

$$\beta_t(m,d) = \begin{cases} \mathcal{S}^*_{t+1}(m)\psi^*_t(m), & d=1 \\ \beta_{t+1}(m,d-1)\psi^*_t(m), & d>1 \end{cases} \tag{47}$$

   where, again,

$$\mathcal{S}^*_{t+1}(m) = \sum_n \mathcal{E}^*_{t+1}(n) A(n,m) \tag{48}$$

$$\mathcal{E}^*_{t+1}(n) = \sum_d \beta_{t+1}(n,d) p_n(d) \tag{49}$$

4. Use the above equation to calculate $\mathcal{E}^*_t$ and $\mathcal{S}^*_t$. Save $\mathcal{E}^*$.

1.5.5 Sufficient Statistics

Finally, we can calculate and return:

$$\log Z = -\sum_{t=1}^{T} \log r_t \tag{50}$$

$$\xi_t(m) = \sum_d \alpha_{t|T}(m,d) = \gamma_{t|T}(m) = \sum_d \alpha_{t|t-1}(m,d)\beta_t(m,d) \tag{51}$$

$$\Xi_{t+1,t}(n,m) = \mathcal{T}_{t+1|T}(n,m) = \mathcal{E}^*_{t+1}(n) A(n,m) \mathcal{E}_t(m) \tag{52}$$

$$C_t(m,d) = \mathcal{D}_{t|T}(m,d) = \begin{cases} \pi_m p_m(d) \beta_1(m,d) & t=1 \\ \mathcal{S}_{t-1}(m) p_m(d) \beta_t(m,d) & t>1 \end{cases} \tag{53}$$

Note that, because $\Xi_{t+1,t}$ is the *joint* absolute probability of the transition (i.e., not conditioned on $d_t = 1$) it is *not* normalized when summing over m and n. The same is true for C when summing over m and d.

2 Inference

2.1 Conjugate updates

For updates on the overdispersion, firing rate, and hyperparameter variables, we have the simple conjugate update rules depicted in Table 2. These can be derived either from free-form variational arguments, or exponential family rules, but are in any case straightforward [5]. If we assume a $\text{Ga}(a,b)$ prior and $\text{Ga}(\alpha,\beta)$ variational posterior, all of these have a simple algebraic update in terms of sufficient statistics.

Here, we overload notation to write $N_{tu} = \sum_m \delta_{u(m),u}\delta_{t(m),t} N_m$, $\overline{\theta}_u = \sum_m \delta_{u(m),u}\overline{\theta}_m$, and make use of H, G, and F as defined in Eq (7) - (9). Indeed, our implementation caches F and G for a substantial speedup (at the cost of additional memory requirements). For autocorrelated noise, the update rules are for the j-th item in the series of autocorrelation.

Table 2. Conjugate updates for Gamma distributions

**Variable**	$\alpha - a$	$\beta - b$
θ_m	N_m	$H_{0u(m)} F_{t(m)u(m)} G_{t(m)u(m)}$
s_u	$\frac{1}{2} \sum_m \delta_{u(m),u}$	$\sum_m \delta_{u(m),u} [\overline{\theta_m} - \overline{\log \theta_m} - 1]$
λ_{0u}	$\sum_t N_{tu}$	$\overline{\theta}_u H_{0u} \sum_t F_{tu} G_{tu}$
λ_{zuk}	$\sum_t N_{tu} \xi_{tk}$	$\overline{\theta}_u H_{0u} \sum_t F_{tuk} G_{tu}$
λ_{xur}	$\sum_t N_{tu} x_{tr}$	cf. Section 2.2
c_0	$U/2$	$\sum_u \mathbb{E}_q \left[d_0 \lambda_{0u} - \log \lambda_{0u} - \log d_0 - 1\right]$
c_{zk}	$U/2$	$\sum_u \mathbb{E}_q \left[d_{zk} \lambda_{zuk} - \log \lambda_{zuk} - \log d_{zk} - 1\right]$
c_{xr}	$U/2$	$\sum_u \mathbb{E}_q \left[d_{xr} \lambda_{xur} - \log \lambda_{xur} - \log d_{xr} - 1\right]$
d_0	$U\mathbb{E}_q[c_0]$	$\sum_u \mathbb{E}_q \left[c_0 \lambda_{0u}\right]$
d_{zk}	$U\mathbb{E}_q[c_{zk}]$	$\sum_u \mathbb{E}_q \left[c_{zk} \lambda_{zuk}\right]$
d_{xr}	$U\mathbb{E}_q[c_{xr}]$	$\sum_u \mathbb{E}_q \left[c_{xr} \lambda_{xur}\right]$
ϕ_{ju} for $j \in \{1, \ldots, \tau\}$	$\sum_{\tau' \geq j} N_{\tau' u}$	$\sum_{u, \tau \geq j} F_{t(\tau)} \prod_{\substack{\tau' \leq \tau \\ \tau' \neq j}} \frac{\omega_{\tau' u}}{\zeta_{\tau' u}}$

2.2 Non-conjugate updates

We employ explicit optimization steps for two updates in our iterative algorithm. In each case, we employ an off-the-shelf optimization routine, though more efficient alternatives are likely possible.

2.2.1 Covariate firing rate effects

Because we do not restrict the covariates $x(t)$ to be binary, Equation 6 no longer yields a conditional log probability for λ_x in the exponential family. This leaves us with a transcendental equation to solve for β_{xr}. However, from Table 2, we see that $\alpha_{xr} \gg \sum_t x_{tr}$, allowing us to approximate $G_t \approx \prod_r (\alpha_{xr}/\beta_{xr})^{x_{tr}}$ as we have done above. Moreover, since the sum $\sum_t G_t \sim T\overline{G}$, we expect $\alpha_x/\beta_x \approx 1$ at the optimum for most reasonable datasets. We thus reparameterize $\beta_{xur} = \alpha_{xur} e^{-\epsilon_{ur}}$ and write the relevant ϵ-dependent piece of the objective function $\mathcal{L}$ as

$$\mathcal{L}_\epsilon = \sum_{ur} \left[a_{xur} \epsilon_{ur} - b_{xur} e^{-\epsilon_{ur}}\right] - \sum_m \overline{\theta}_m H_{0u(m)} F_{t(m)u(m)} e^{-\sum_r \epsilon_{u(m)r} x_{t(m)u(m)r}} \quad (54)$$

We also supply the optimizer with the gradient:

$$\nabla_\epsilon \mathcal{L}_\epsilon = a_{xur} - b_{xur} e^{-\epsilon_{ur}} - \sum_{m; u(m)=u} x_{t(m)ur} \overline{\theta}_m H_{0u} F_{t(m)u} e^{-\sum_r \epsilon_{ur} x_{t(m)ur}} \quad (55)$$

where we sum only over observations with $u(m) = u$. On each iterate, we then optimize $\mathcal{L}_\epsilon$, initializing β_x to the just-updated value of α_x. In addition, we do not update firing rate effects for covariates separately, but optimize β_{xur} for all r together. Again, more efficient schemes are no doubt possible.

2.3 Semi-Markov duration distribution

If the dwell time distributions of states in the semi-Markov model were truly continuous, the parameters (m, s^2) of a lognormal distribution $p(d|j)$ would have a Normal-Gamma conjugate prior, and updates would be closed-form. However, the requirement that the durations be integers and that there exist a maximal duration, D, over which these probability mass functions must normalize results in an extra term in $\mathbb{E}_q[\log p]$ arising

from the normalization constant. In this case, we must explicitly optimize for the parameters of the Normal-Gamma prior on (m, s^2). However, unlike the case of 2.2.1, these parameters are only updated for one latent feature at a time. Since the Normal-Gamma distribution has only 4 parameters and the number of states of the latent variable is $S = 2$, this requires updating $4SK$ parameters, but only taken $4S = 8$ at a time, a much more manageable task.

3 Experiments

Code for all algorithms and analyses is available at `https://github.com/pearsonlab/spiketopics`.

References

1. Beal MJ. Variational algorithms for approximate Bayesian inference. University of London; 2003.

2. Yu SZ, Kobayashi H. Practical implementation of an efficient forward-backward algorithm for an explicit-duration hidden Markov model. Signal Processing, IEEE Transactions on. 2006;54(5):1947–1951.

3. Mitchell CD, Jamieson LH. Modeling Duration in a Hidden Markov Model with the Exponential Family. In: Acoustics, Speech, and Signal Processing; 1993.

4. Mitchell C, Harper M, Jamieson L. On the complexity of explicit duration HMM's. IEEE Trans Audio Speech Lang Processing. 1995;3(3):213–217.

5. Blei DM, Jordan MI. Variational inference for Dirichlet process mixtures. Bayesian Anal. 2006;1(1):121–143.
