# Supplementary material for: Neuron’s eye view: Inferring features of complex stimuli from neural responses
Source: PLoS Comput Biol. 2017 Aug 21;13(8):e1005645. doi: 10.1371/journal.pcbi.1005645 (PMC5578681; doi:10.1371/journal.pcbi.1005645)
Supplement: S2 Text — (PDF) [file pcbi.1005645.s002.pdf]

S2 Text. Inferred latents as classification features

As a byproduct of our model, the generated labels provide a concise and fairly complete summary of the stimulus-related activity in the neural recordings. Here we want to emphasize that although our model is not a data compression method, it nonetheless preserves most of the information from raw data.

Here, we use all pairwise combinations of binary features of our model to train a sparse logistic regression predicting stimulus category. We compare the results of this to a multinomial logistic regression on raw spike counts with L1 and L2 regularization. Results are shown in Figure 1.

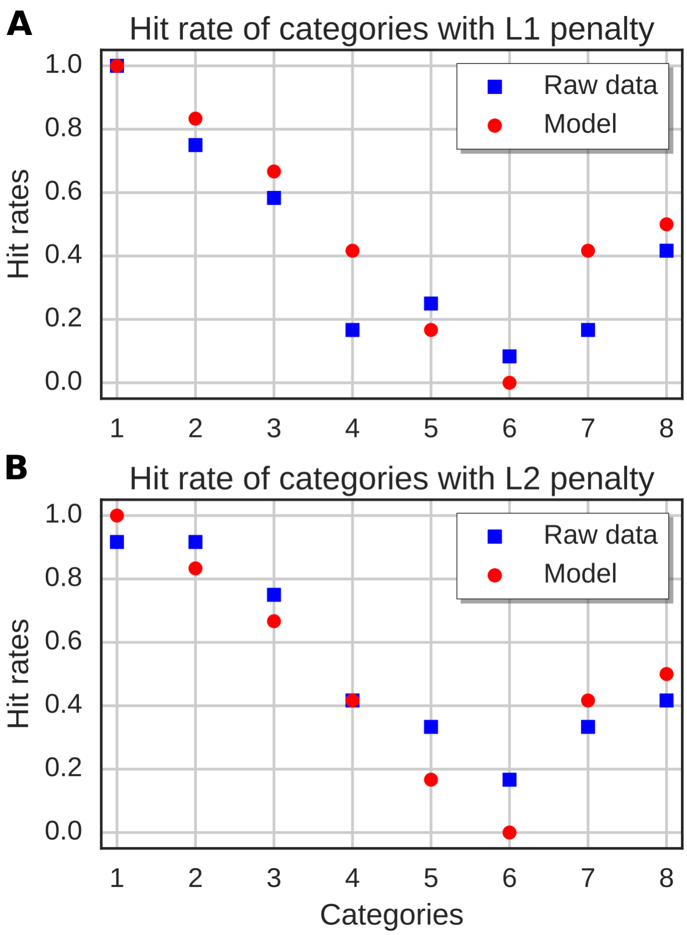

Figure 1. Comparison of actual and inferred states of the macaque dataset. A. The overall hit rate of prediction with L1 regularization respect to eight categories: Faces, Animals, Bodies, Fruit, Natural, Manmade, Scene, Pattern. B. The overall hit rate of prediction with L1 regularization for eight categories.
