# Supplementary material for: Neuron’s eye view: Inferring features of complex stimuli from neural responses
Source: PLoS Comput Biol. 2017 Aug 21;13(8):e1005645. doi: 10.1371/journal.pcbi.1005645 (PMC5578681; doi:10.1371/journal.pcbi.1005645)
Supplement: S3 Text — (PDF) [file pcbi.1005645.s003.pdf]

S3 Text. Effects of bin size and dynamics

Robustness of inference to time step size

Our model assumes a fixed time step Δt between count observations, which is also the time step for the Hidden Markov Model. However, if the time step used for inference (and binning spike data) is too large, the model may miss important temporal features of the data. Here, we performed two experiments to test the robustness of our inferred features to these possibilities. In both cases, we simulated data from our model ($U = 20$ units, $T = 1$s stimulus length, $M = 200$ trials, $\Delta t = 5$ms, $K_{data} = 3$ features, $K = 5$ inferred features), similar to our synthetic data experiment. Just as in that experiment, our goal was to compare our recovered latent features to those that generated the data. However, in our inference, we systematically varied $\Delta t = (5, 10, 20, 50, 100)$ ms. We used the same relative error tolerance for training as in our synthetic data experiment, with 10 random parameter restarts, from which we chose the solution with highest ELBO. We did not include overdispersion effects in this experiment, though similar results may be obtained in that condition by including a larger number of trials.

Results are displayed in Figure 1. At the top, we display the binary features used to generate the data. Below this, we show recovered binary features for each bin size, weighted by the mean absolute effect size (as a percent change from baseline) across the units in our simulation. This weighting serves to make inferred features with negligible effect on firing less visually salient, since a feature may be inferred with a low accompanying firing rate, effectively rendering it unused. Clearly, even though some discovered features are inverted, the model faithfully recovers the latent dynamics for small bin sizes (up to 20ms), gracefully degrading for larger bin sizes. This effect is likewise obvious in the comparison of actual, empirical, and recovered firing rates in Figure 2, where deviations become more apparent as the time step increases and the model's recovered firing becomes coarser.

Robustness of inference to firing rate dynamics

As a second check of model robustness, we repeated the bin size experiment of the previous but added a transient increase in firing rate (e.g. stimulus onset response) to the beginning of each trial. This transient was encoded as an additional multiplicative effect of $1 + 0.1\Gamma(5, 80)$, where $\Gamma(a, b)$ is the pdf of the Gamma distribution (in the shape, rate parameterization) and the data generating Δt was again 5ms.

Figure 3 shows the results in the same format as Figure 1. Here, the model correctly recovers the underlying features for small time bin sizes, accounting for the transient by utilizing extra features (3 and 4) in the model. That is, in this simplified scenario, the model's response to additional temporal dynamics is simply to infer additional features. Nevertheless, as in the previous section, with larger time steps, features begin to blend, and the transient dynamics is poorly captured. This is also visible in the corresponding firing rate reconstruction (Figure 4)

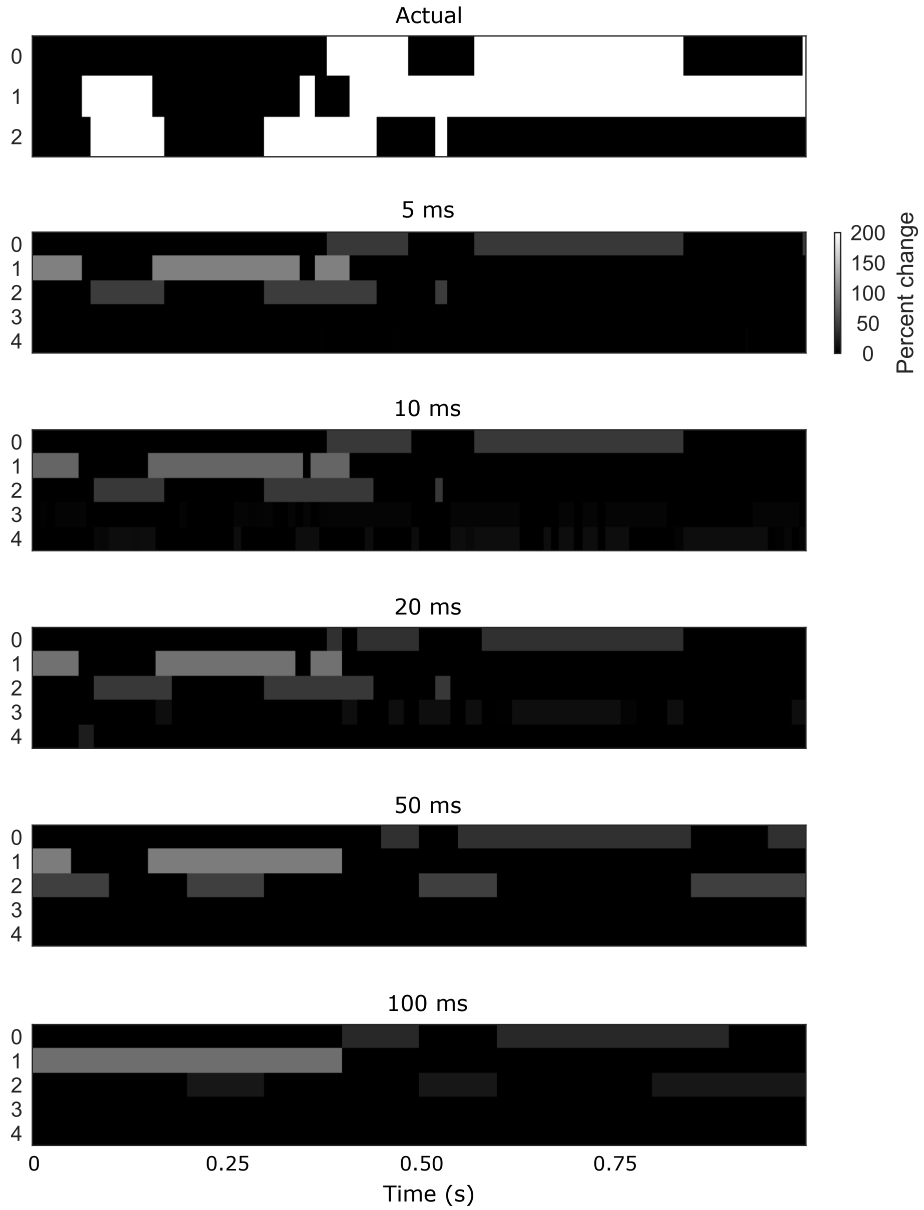

Figure 1. Recovery of latent features with different time step sizes. Comparison of recovered latent features from synthetic data generated with a 5ms time step resolution. Top panel shows actual binary features, while bottom panels show recovered features for a range of increasing time step sizes. Recovered features are plotted as $\mathbb{E}[z]|\mathbb{E}[\lambda]-1|$, the expected value of the binary feature weighted by the absolute magnitude of the effect (calculated as a fractional change in firing rate from baseline). That is, brighter colors indicate features corresponding to larger changes in firing rate, both positive and negative.

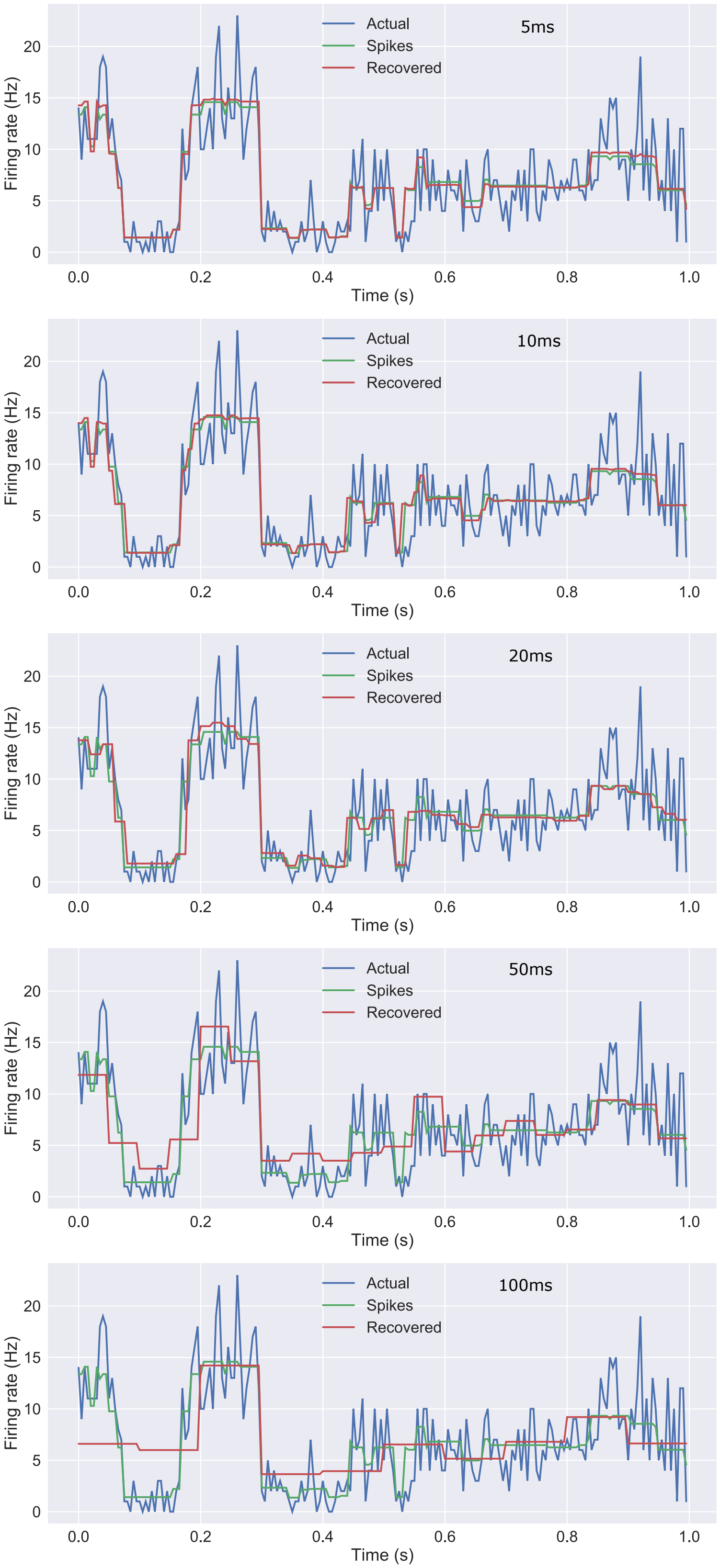

Figure 2. Recovery of firing rates with different time step sizes. Comparison of actual firing rates, trial-averaged empirical firing rates, and model-recovered firing rates for a variety of time step sizes.

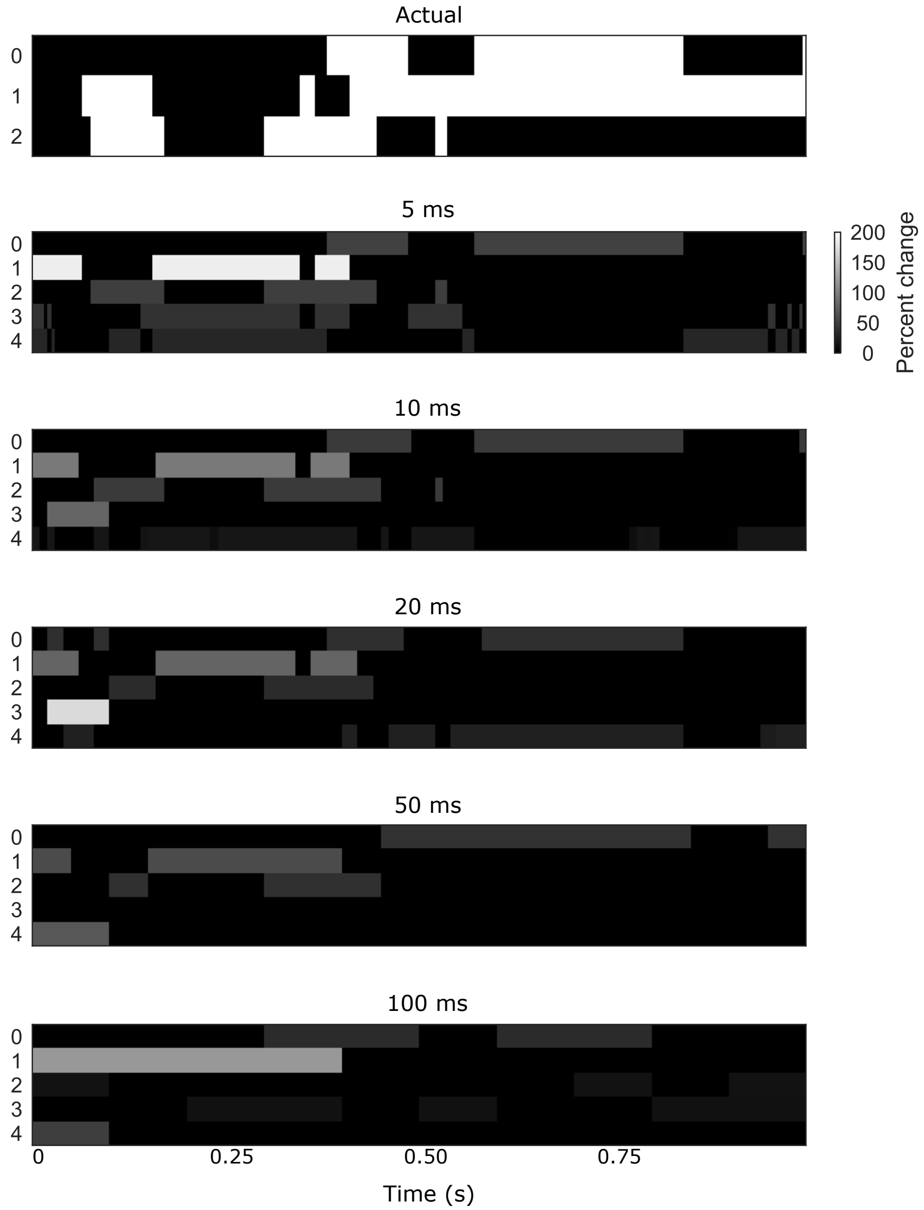

Figure 3. Recovery of latent features with transient dynamics. Comparison of recovered latent features from synthetic data generated with a 5ms time step resolution and a transient increase in firing at the start of each trial. Top panel shows actual binary features, while bottom panels show recovered features for a range of increasing time step sizes. Color scale is as in Figure 1.

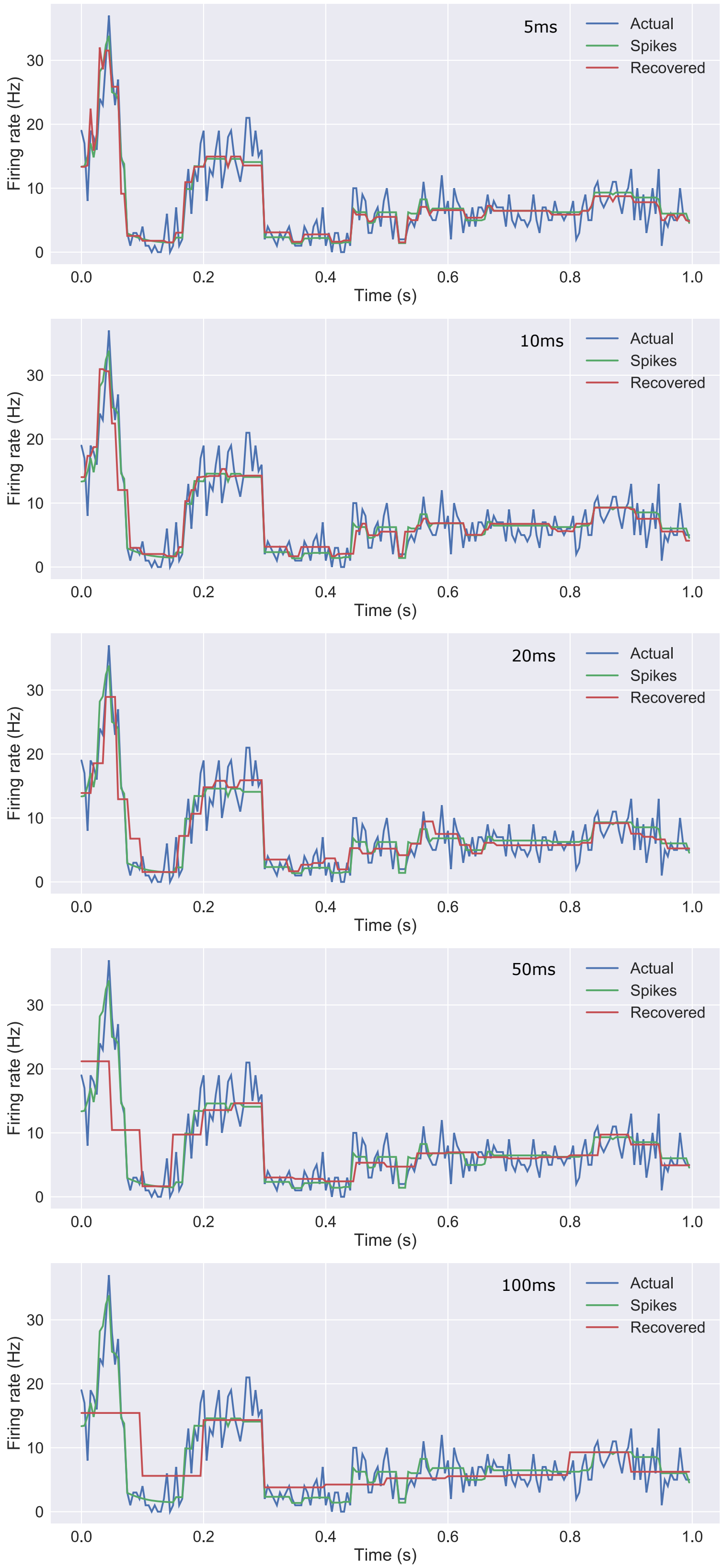

Figure 4. Recovery of firing rates with transient dynamics. Comparison of actual firing rates, trial-averaged empirical firing rates, and model-recovered firing rates for a variety of time step sizes. Cf. Figure 2, which shares the same latent dynamics but lacks the transient.
